# Bayesian statistics in anesthesia practice: a tutorial for anesthesiologists

**DOI:** 10.1007/s00540-022-03044-9

**Published:** 2022-02-11

**Authors:** Michele Introna, Johannes P. van den Berg, Douglas J. Eleveld, Michel M. R. F. Struys

**Affiliations:** 1 Department of Anesthesiology, University Medical Center Groningen, University of Groningen, Hanzeplein 1, 9713 GZ Groningen, The Netherlands; 2 Department of Anesthesiology and Intensive Care Medicine, Cremona Hospital, Cremona, Italy; 3 Department of Basic and Applied Medical Sciences, Ghent University, Ghent, Belgium

**Keywords:** Bayesian, Statistics, Clinical research, Anesthetic pharmacology

## Abstract

This narrative review intends to provide the anesthesiologist with the basic knowledge of the Bayesian concepts and should be considered as a tutorial for anesthesiologists in the concept of Bayesian statistics. The Bayesian approach represents the mathematical formulation of the idea that we can update our initial belief about data with the evidence obtained from any kind of acquired data. It provides a theoretical framework and a statistical method to use pre-existing information within the context of new evidence. Several authors have described the Bayesian approach as capable of dealing with uncertainty in medical decision-making. This review describes the Bayes theorem and how it is used in clinical studies in anesthesia and critical care. It starts with a general introduction to the theorem and its related concepts of prior and posterior probabilities. Second, there is an explanation of the basic concepts of the Bayesian statistical inference. Last, a summary of the applicability of some of the Bayesian statistics in current literature is provided, such as Bayesian analysis of clinical trials and PKPD modeling.

## Introduction

The Bayes theorem was developed over 250 years ago by Bayes [1], a Presbyterian minister. For a long era the “Bayesian” approach to inferential statistics has been dominated by the “frequentist approach”. However, since the 1980s, there has been a renewed interest in the possibilities offered by the applications of this theorem in many fields of medical research, including anesthesiology [2–4]. In contrast to the more conventional frequentist approach which focuses on the frequency that an event occurs, the Bayesian approach is concerned with the uncertainty about data. As such it is a very useful technique for biostatistics and is commonly applied in anesthetic literature. This recent renaissance can challenge readers with complex literature, which can be difficult to understand for clinicians. This review introduces the reader to the basics of the Bayesian framework and how this can be encountered in scientific research in the field of anesthesia.

## The frequentist and Bayesian approach

The process of gaining knowledge from experience follows a consistent pattern. The crucial step is to assess whether evidence from clinical trials, experiments, clinical data, etc. is strong enough to change an a priori belief about any phenomenon or whether the a priori belief remains unchanged. The Bayesian approach provides a theoretical framework and a mathematical method to use pre-existing information within the context of new evidence.

The well-established frequentist statistical methods are based on the process of estimating the characteristics (or parameters) of a population, by the analysis of a randomly selected sample from this population. As such, with an increasing number of samples, the relative change of the estimation of parameters will decline. This approach has been historically called “frequentist”, because the process of inference demands many repeated events.

Under the frequentist approach, the “true” parameters to be estimated in the population are fixed, but unknown. The estimation of such parameters can be done through a sampling of the population. This generates a distribution of estimates that quantifies how uncertain we are about them. A significant difference between two groups regarding a parameter implies that the difference in the distribution of the estimates is unlikely to come by chance alone. The result of statistical frequentist tests that compare quantities in two or more groups generates a normal distribution of the differences and calculates a *P* value (probability value), confronted with a pre-specified cutoff value. “The *P* value tells us how likely it would be to observe results at least as extreme as what we saw in our study if the null hypothesis is true” (${\mathrm{H}}_{0}$, that there is no real difference between groups) [5]. Like in any diagnostic test, a positive result and the knowledge of its sensitivity or specificity does not provide any information regarding the probabilities of finding the disease. In the same way, the *P* value cannot give any statement regarding the probability that the results of the trial are representative of the underlying reality [6]. These misconceptions are known as “base rate fallacy” [7]. The *P* value should be interpreted as the clinician interprets the result of a test in light of the pre-test probabilities [8]. It should be calibrated with respect to the probabilities that the test result does not represent a first or second type error (alpha and beta), not provided by the *P* value per se. Unfortunately, in the frequentist approach it is not possible to explicitly include this information in the statistical calculation.

The above-mentioned issues with some aspects of the frequentism have motivated researchers and statisticians to find other ways of analyzing their data and expressing the results. The Bayesian approach to statistical inference has some unique aspects that can be of use to address these issues. Knowing the probability of a hypothesis given the observed results (combined with information prior to the trial itself) could be more useful for the clinicians. This is the reason why there has been a substantial increase in the interest of the Bayesian techniques in data analysis in recent years [9]. However, several authors have expressed concerns regarding the application of Bayesian inference in the clinical decision-making process. Probabilistic reasoning involves mental processing of probabilities in a single event format (e.g., 1% probability). This type of modality may be less intuitive in the clinician’s mind than reasoning based on event rates (e.g., 1 event in 100) [10]. For that reason, in a setting of time-pressured medical decisions, the probabilistic reasoning can be challenged by heuristics and cognitive bias, leading to errors. Considering this, there is a growing need for probability reasoning tools and Bayesian inference principles, to help the clinicians in their correct application [11]. This review has been developed to introduce the anesthesiologist to the Bayesian framework, emphasizing its advantages and clarifying its limitations.

Thus, the limitation of the frequentist approach resides in its logical inconsistency with the clinical decision-making process, if not correctly interpreted. On the other hand, the Bayesian approach logic is intrinsically consistent with the inductive nature of the clinical reasoning, but it suffers from other limitations, addressed in the following paragraphs (e.g., the subjective choice of the priors). It is important to understand and correctly interpret both the frequentist and Bayesian approaches, each with advantages and drawbacks, as summarized in Table 1.

**Table 1:** Comparison of the frequentist and the Bayesian approach

	Frequentist	Bayesian
Answer given	The probability of the observed data given an underlying (unknown) truth	The probability of the underlying truth given the observed data
Population parameter	Fixed, but uknown	Probability distribution of values (quantifying uncertainty)
Outcome measure	The probability of observing results at least as extreme as the study data, assuming true the null hypothesis (*P* value)	The posterior probability of the hypothesis
Weaknesses	Logical inconsistency with the clinical decision-making process	Subjectivity in the priors’ choice; non-traditional methods (statistical complexity); does not always work well during rapid changes in PKPD modeling
Strengths	No need for priors (objectivity); traditional, well-known methods	Consistency with the clinical (inductive) decision-making process
PKPD application	Good estimates with large quantity of data (population)	Adaptation of population data to the single individual through feedback systems

In the table, an overview of the differences, strengths, and limitations of both the frequentist and the Bayesian approach is given.

## The conditional and inverse probability

**Fig. 1:**
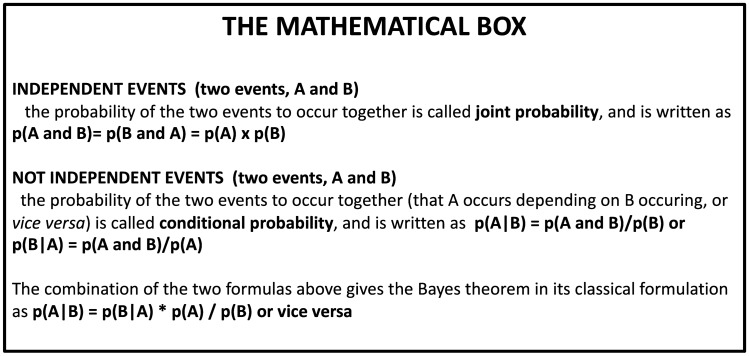
The mathematical box

Two essential mathematical concepts are essential to understand the Bayesian theorem: the conditional and inverse probability. A conditional probability is the probability of an event A occurring given that another event B occurred. In mathematical notation, this is *p*(*A*|*B*). In a deck of 52 cards, the probability of getting a queen (event A) given that someone drew a black card (event B) is noted as *p*(Queen|black). There are 26 black cards, so the conditional probability is equal to the number of times black cards with a queen divided by the total number of black cards in the deck: *P*(Queen|black) = 2/26. The probability of event A can therefore depend on whether event B was observed or not. The Bayes theorem is a formula (Eq. 1) that permits calculating the reverse of a conditional probability. In our example, it answers the reverse question, namely the probability of getting a black card given that someone drew a queen. Formula (2) describes our example. The mathematical derivation of the formula can be found in the mathematical box below (Fig. 1).

1 $$P\left(A|B\right)=\frac{P\left(B|A\right)*P\left(A\right)}{P\left(B\right)},$$

2 $$p\left( {{\text{black}}|{\text{Queen}}} \right) = \frac{{P\left( {{\text{Queen}}|{\text{black}}} \right)*P\left( {{\text{black}}} \right)}}{{P\left( {{\text{Queen}}} \right)}} = \frac{{\frac{2}{{26}}*\frac{{26}}{{52}}}}{{\frac{4}{{52}}}} = {\raise0.7ex\hbox{$1$} \!\mathord{\left/ {\vphantom {1 2}}\right.\kern-\nulldelimiterspace} \!\lower0.7ex\hbox{$2$}}.$$

Sometimes, in real-world cases, the *P*(*B*) term is quite difficult to obtain. The most common form of the Bayes theorem is, therefore, *P*(*A*|*B*) α *P*(*B*|*A*) * *P*(*A*), using *P*(*B*) as a constant of proportionality. This formula can be applied whenever a probability calculation is involved, like the probability of the observed evidence to be true given any a priori belief, or the inverse, using the formula above.

This could be further explained using a clinical example, such as a laboratory blood result in the preoperative screening, most likely in asymptomatic patients. As such, tests could be false positive. Every test has a known value of sensitivity, which reflects the proportion of true positives out of the total of the positives, being false or true. It also has a value of specificity, which indicates the percentage of true negatives out of the sum of the negatives. Therefore, sensitivity reflects the probability of evidence (the positive test) in the light of a hypothesis (the patient has a disease). In mathematical terms, it is called conditional probability, and it is written as *P* (positive test|hypothesis). On the other hand, it is relevant to know the probability of a hypothesis considering the evidence. The critical factor that influences the interpretation of the results would be the prevalence of the disease in the patient population, not only the specificity or the sensitivity of the test. If the patient was a young athlete undergoing surgical treatment for an inguinal hernia, the prevalence of an actual disease could be very low, which may differ from a frail, older patient.

## The Bayes theorem explained

### Hypothesis, prior and posterior probability

The Bayes theorem is expressed in formula (3). It derives by expressing the mathematical relationships of two elements (e.g., black and queen in the above-mentioned example). These two elements are the prior belief about a phenomenon (e.g., the hypothesis about the effects of a treatment) and the data from the evidence (e.g., a clinical trial). Note that $P\left(\mathrm{data} \right| \mathrm{belief})$ describes the knowledge about how the evidence would be possible if the evidence were true, also known as likelihood. Instead, $P\left(\mathrm{belief}\right)$ describes the prior and $P \left(\mathrm{belief} \right| \mathrm{data})$ represents the posterior (output) probability.

3 $$P \left(\mathrm{belief} \right| \mathrm{data}) \alpha P\left(\mathrm{data} \right| \mathrm{belief}) P\left(\mathrm{belief}\right).$$

The key concept of the Bayesian approach is calculating the posterior probability as a result of observed data and the prior beliefs about that data. This is proportional (*α*) to the likelihood of the data, given the hypothesis and the prior probability of the hypothesis. This concept can be illustrated by means of an example, such as the anticipation of difficult intubation.

The anesthesiologist often uses information that is acquired before (or prior) the procedure (e.g., preoperative tests such as a structured physical examination and Mallampati score and medical history). [12, 13]. However, during intubation the clinician can decide to change his/her strategy based on new unfolding evidence [14]. Here, the hypothesis is the probability of successfully intubating the patient, while the “data” are the pieces of evidence collected by the anesthesiologist before the start of the procedure that are relevant to the accessibility of the patients airways. The Bayes theorem’s central significance in this context is to update the posterior chances of successful intubation by adding newly found information. This concept of ‘probabilistic’ thinking is the corner stone of the Bayes theorem, which is at the same time its strength and weakness. The strength of the prior depends on how much weight it is given (e.g., assessment by a junior resident might provide a weaker prior than when it is performed by a senior consultant). Those priors can therefore be expressed as probability distributions describing the uncertainty around a certain parameter (e.g., the treatment effect). The choice of the distribution of the prior probabilities is probably the most challenging and criticized component of any Bayesian analysis. The most often cited concern is the “subjectivity” that the choice of the priors introduces in the analysis. There are different approaches for dealing with the subjective nature of prior distributions, such as the use of uninformative or evidence-based priors. On the contrary, the absence for a prior choice has historically given the opportunity to the frequentist approach of being considered more objective.

### Uninformative and evidence-based prior

There can be circumstances in which there is only limited prior information. In our proposed example, this occurs before any physical examination has taken place and the patient presents in an emergency situation requiring immediate endotracheal intubation. These priors are referred to as “uninformative”. It is typically handled by assigning the same probability for every relevant prior, with the risk of being overwhelmed by the new data. In broad terms, “flat” or “non-informative” priors are the ones that add little influence to the posterior distribution (e.g., the intubation without adequate preoperative evaluation), with the effect of producing results mostly dependent on the data available (the likelihood distribution).

In contrast to “uninformative” priors, there is also the possibility that data are already available about the scientific question. Then an alternative method of choosing the priors can be considered by including in the analysis the data from relevant studies. This second strategy is called “evidence-based prior”.

These different ways of considering the prior beliefs that the researchers could have regarding the outcome comprise the potential advantage of the Bayesian approach, but these can also be considered as its Achilles’ heel [15]. To address some issues in the interpretation of the prior probabilities, several guidelines are available. The reader can find useful references in the review by Houle, “Bayesian Statistical Inference” in Anesthesiology [2]. The priors must be explicitly reported, justified, and subjected to a sensitivity analysis.

### Iterative use of Bayes’ theorem

The elegance of the Bayesian theorem is that it can be applied in an iterative manner since the posterior probability from any calculation can serve as a prior for a subsequent calculation [16]. The result is a refined set of data from the entire experience. This can also be used to incorporate the probability distribution of the priors with the likelihood derived from the experiment to get a posterior distribution of the outcomes.

The ability to use Bayes theorem even when the starting point was affected by uncertainty is an important argument for the successful use in many situations.

## The Bayes theorem and statistical inference

**Fig. 2:**
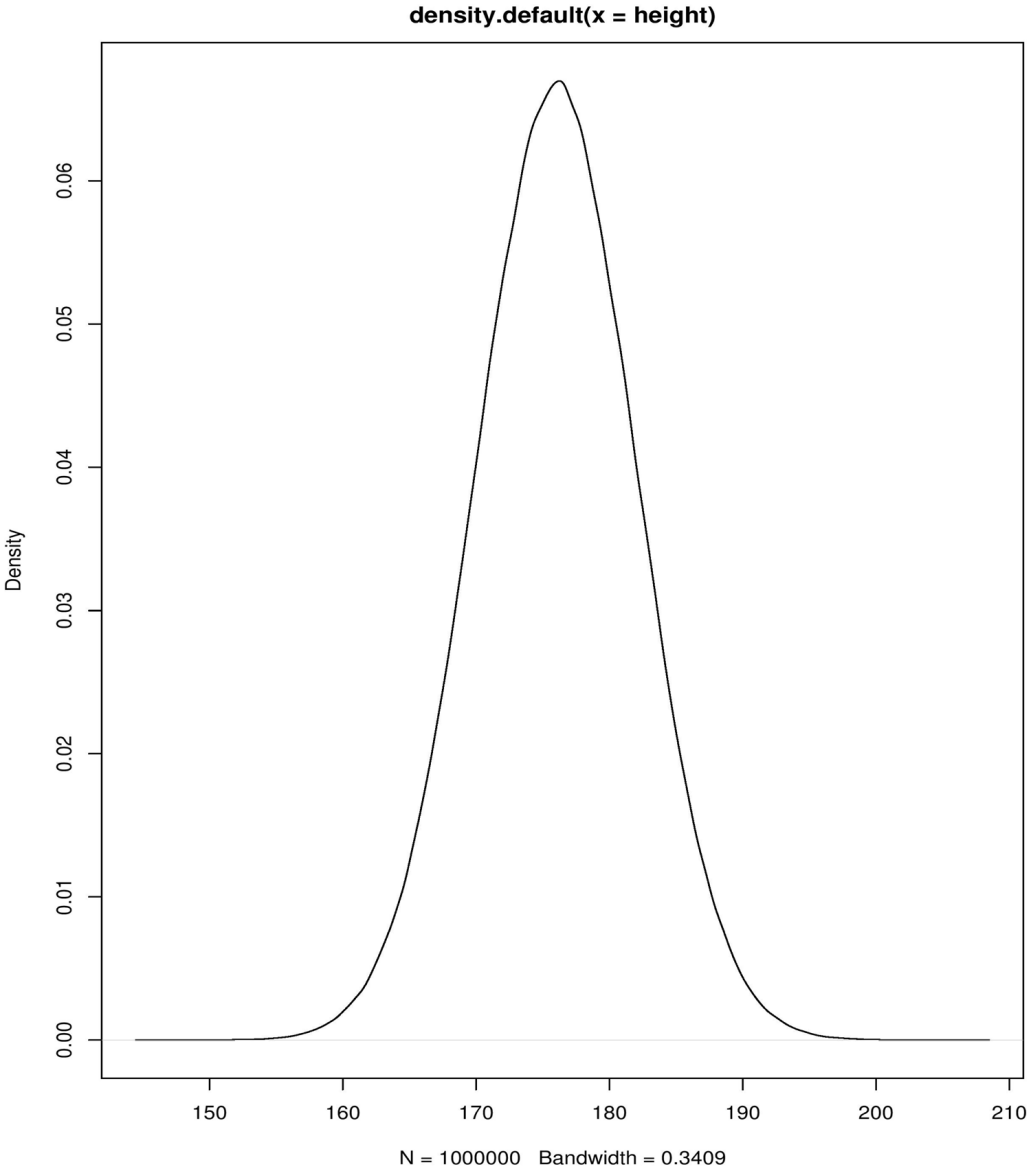
The probability distribution

The clinician is often confronted with contradicting trial results which address questions about the probability of effectiveness of an alternative therapy, the strength of the available evidence and how this new information fits the results of previous trials [17]. These concerns are not that different from the considerations regarding the success of an intubation attempt and depend on the likeliness of a claim to be true given the trial results and the priors. The Bayes approach to statistical inference can provide a mathematical framework to answer these questions. Contrary to the frequentist approach in which parameters are fixed, but unknown, in the Bayesian framework the parameters are treated as random variables that are subject to a probability distribution (Fig. 2). A probability distribution curve describes the probability of the variable to fall within a particular interval [18]. The most commonly encountered example is the Gaussian or “normal” distribution. Instead of calculating the probability of any possible outcome given an underlying unknown truth, the Bayesian approach calculates the probability of the underlying truth given the observed data [19, 20]. The probability distribution of the priors is mathematically combined with the likelihood (the probability of the observed data given the parameters) to generate a posterior distribution of the outcomes. This posterior distribution (see below) is more informative for a clinician in terms of expected chances of the outcome than a cutoff level of statistical significance (i.e., the chances that a certain treatment exerts a pre-specified effect).

In the graph, the typical example of the probability distribution for heights in the adult population (mean 176 cm, SD 6 cm, *N* = 1,000,000 simulated data points, software R studio 3.5.1) is depicted. It is a probability distribution because when any interval is chosen, the integral function (i.e., the area under the curve) calculates the probability that a random value from the population lies within this interval. For example, the probability of a random individual to be taller than 190 cm is calculated as the AUC of the curve from *x* = 190 to *x* = infinite. The variance of the distribution is represented by the width of the distribution and is indicative of the certainty of the outcome considered (in this example, we could have a very short and wide peak, meaning scattered, uncertain data).

## Understanding a Bayesian paper in anesthesiology

We aim to illustrate the difference between the “Bayesian” and “frequentist” approach on the basis of a recent paper published in British Medical Journal Open [21]. This is a post hoc Bayesian analysis of the OPTIMISE study, which is designed as a pragmatic, multicenter, observer blinded, randomized controlled trial involving 734 high-risk patients undergoing major gastrointestinal surgery in 17 acute care hospitals in the UK [22]. Patients were randomly assigned (1:1) to a cardiac output-based goal-directed hemodynamic therapy algorithm for intravenous fluid and inotropic drug administration (intervention; *n* = 368) or to standard care (*n* = 366). The study duration was the 6 h following surgery. The primary outcome was a composite of predefined 30-day moderate or major complications and mortality. The OPTIMISE study “was not powered to detect a statistically significant difference between the treatment arms for the observed side effect” (relative risk = 0.84, 95% CI 0.7–1.01; *P* value = 0.07). As such, there was no statistically significant difference between the two groups in the standard “frequentist” analysis. The re-analysis by Ryan et al. was based on the idea of an alternative, Bayesian-based tool for clinicians in interpreting the trial data.

In the case of the work by Ryan and coworkers, the two conventional methods for the prior choice explained above were used. The first one was to consider a “flat prior”, which implies an equal probability for every value of the relative risks. The second one was the “evidence-based prior”, including data from relevant studies from a meta-analysis [22, 23].

In the Ryan et al. analysis, the posterior mean relative risk (RR), resulting from the prior distribution that is updated with the trial data under the flat prior, is 0.85 (95% high density interval (HDI) 0.70–1.00). As this paper suffers from weaker priors, the strength of the data from the trial would give similar results to the original frequentist analysis. Using the evidence-based priors instead, the posterior mean RR is 0.81 (95% HDI 0.67–0.95). Making use of the posterior probability distribution of RR obtained from the Bayesian analysis, the first conclusion the authors drew is that the probability that the intervention group has a lower incidence of 30-day moderate or major complications and mortality is 96.9% and 99.5%, assuming a flat and evidence-based prior, respectively. It is now clear how different the interpretation of the trial data could be through a Bayesian analysis. Through the conventional approach, the difference between the groups is not sufficient to generate an answer that is useful to the clinicians. Instead of an absence of evidence represented by a non-significant *P* value (especially in the case of an underpowered trial), the authors claim instead that the probability of an effect on the outcomes is quite high (more than 90%). This approach could be directly informative for treatment decisions bedside, more than a cutoff level of statistical significance. The advantage of the Bayesian approach is that if the priors and posteriors are correctly reported, there is room for open discussions on specific elements in the analysis.

Along with this Bayesian analysis, the authors introduce the concept of region of practical equivalence (ROPE). This tool is easily understandable for the clinicians. It represents the probability that the effects lay outside a specific interval of clinically insignificant results. With the ROPE, the authors define a region of values on the probability distribution curve of the RR that are practically equivalent to the null value [24]. In this particular study, the ROPE of the effects were pre-specified to be 0.9–1.1 regarding the RR. The probability of the two arms to be clinically equivalent (i.e., within the ROPE) regarding the RR was 24% under the flat priors and 9% under the evidence-based priors, respectively (i.e., 76% and 91% probability of a clinically relevant difference).

## The Bayes theorem in anesthetic pharmacology

Besides the analysis of new insights from literature, the Bayes theorem is also frequently applied in the field of anesthetic pharmacology, especially in pharmacokinetic and dynamic (PKPD) research. In this particular case, PKPD models are commonly used to predict a plasma or effect-site concentration in an individual. These models incorporate specific pharmacologic parameters (i.e., clearances and distribution rates) that are predicted based on population characteristics and covariates (i.e., specific patient characteristics, such as age and gender). As mentioned earlier, the probability is expressed in a probability distribution curve that commonly behaves as a Gaussian curve, which has a mean and a standard deviation. It is unlikely that the real parameter estimate *exactly* matches the mean value. Implementing measurements can shift the prediction toward a more realistic value given the observations, using the Bayesian concept and therefore intent to improve the model’s predictive ability.

Maitre et al. [25] and Motamed et al. [26] showed that Bayesian forecasting improved the predictive performance of alfentanil and rocuronium PK–PD models, respectively. More recently, van den Berg et al. used online Bayesian adaptation of the model to improve the PK accuracy of the Eleveld model for propofol during total intravenous anesthesia (TIVA) using target-controlled infusion (TCI) with propofol [27, 28]. This study showed an improvement in bias, but not precision, probably due to the application of an already reasonably accurate population-based model. Colin and coworkers applied a Bayesian algorithm to improve this PK model based on exhaled propofol concentrations [29]. This contributes to the concept of posology as introduced by Kuck and Egan, which is the study of dose optimization [30]. Although these developments to improve PK are of limited clinical relevance in the field of anesthesia, they can be used in pharmacological fields in which dose optimization is crucial, e.g., when the therapeutic range is small, such as antibiotics or cytostatic agents.

Despite the availability of accurate PK models to provide accurate target-controlled infusions, PK parameters are not generally the relevant target for clinicians. It is not the plasma concentration that a clinician is aiming for, rather they titrate drug concentration to achieve pharmacodynamic end points. As such, it appears to be relevant to focus with drug advisory systems on PD end points. Feedback systems that use measurements as input parameters, such as (semi) closed loop systems, could be helpful in optimizing drug titration [31–39]. In this situation, the population-based response model provides the prior distribution of the model parameter values. The posterior estimated model parameter values are adjusted by the algorithm as observations are made. These reflect the patient’s parameters, expressed as a posterior distribution. The infusion of propofol through a TCI pump is then modified according to the information provided by the controller, the response of the patient is further observed, and the model adjusted again. This system has been demonstrated accurate in different clinical settings: gynecological surgery [34, 36], head and neck surgery [36] and also for intensive care sedation [35]. Further clinical studies are needed to evaluate the clinical impact of such a technology on the optimization of propofol administration regarding the standard of care.

Accurate drug administration models applied in TCI can be very helpful for the reproducibility of accurate drug titration. Adaptation of a population-based E-max model of the relationship between predicted plasma concentration and effect (e.g., electro-encephalographic monitoring) toward the individual, most likely with the application of a Bayesian algorithm (using the population-based model as a prior and real-time measurements as data), might provide useful drug dosing advices. This requires further study.

Bayesian techniques rely on its users’ ability to clearly identify the mechanisms and processes to be able to assign a prior belief to them. This is sometimes difficult in medical sciences due to the gap between simplistic mathematical models and immensely complex biological processes. For example, the compartmental models widely used in pharmacokinetic are often obviously “wrong” for modeling the complex dynamic changes that can occur in the first seconds or minutes after drug administration. However, through Bayesian techniques, also the first minutes of administration can be predicted in a compartmental pharmacokinetic model [40]. For slower physiologic changes, for example during a “maintenance phase” these proposed mechanism are typically simpler and it becomes easier to assign a prior belief for subsequent application of Bayesian techniques.

## Conclusion

This review aimed to describe the application of the Bayesian theorem in several fields of anesthetic clinical research. The posterior distribution of the probabilities of the outcome makes the Bayesian approach very useful for evaluation and quantification of uncertainty [41]. Even in the relative absence of data, models can be structured starting with priors, and these can be iteratively updated through experimental data obtained. This behavior, united with its peculiar characteristic of using probability distributions of the prior and posteriors, makes it ideal to represent uncertain quantities in a model and how they relate to the available data [42].

## References

[1] Bayes T (1763). An essay towards solving a problem in the doctrine of chances. By the late Rev. Mr. Bayes, F. R. S. communicated by Mr. Price, in a letter to John Canton, A. M. F. R. S. Philos Trans R Soc London.

[2] Houle TT, Turner DP (2013). Bayesian statistical inference in anesthesiology. Anesthesiology.

[3] Webb MPK, Sidebotham D (2020). Bayes’ formula: a powerful but counterintuitive tool for medical decision-making. BJA Educ.

[4] Ferreira D, Barthoulot M, Pottecher J, Torp KD, Diemunsch P, Meyer N (2020). Theory and practical use of Bayesian methods in interpreting clinical trial data: a narrative review. Br J Anaesth.

[5] Nuzzo RL (2017). An introduction to Bayesian data analysis for correlations. PM R.

[6] Hadjipavlou G, Siviter R, Feix B (2021). What is the true worth of a P-value? Time for a change. Br J Anaesth.

[7] Nuzzo RL (2015). The inverse fallacy and interpreting P values. PM R.

[8] Held L (2010). A nomogram for P values. BMC Med Res Methodol.

[9] Kalil AC, Sun J (2014). Bayesian methodology for the design and interpretation of clinical trials in critical care medicine: a primer for clinicians. Crit Care Med.

[10] Operskalski JT, Barbey AK (2016). MEDICINE. Risk literacy in medical decision-making. Science.

[11] Henriquez RR, Korpi-Steiner N (2016). Bayesian inference dilemma in medical decision-making: a need for user-friendly probabilistic reasoning tools. Clin Chem.

[12] El-Ganzouri AR, McCarthy RJ, Tuman KJ, Tanck EN, Ivankovich AD (1996). Preoperative airway assessment: predictive value of a multivariate risk index. Anesth Analg..

[13] Roth D, Pace NL, Lee A, Hovhannisyan K, Warenits A-M, Arrich J, Herkner H (2018). Airway physical examination tests for detection of difficult airway management in apparently normal adult patients. Cochrane Database Syst Rev.

[14] Frerk C, Mitchell VS, McNarry AF, Mendonca C, Bhagrath R, Patel A, O’Sullivan EP, Woodall NM, Ahmad I (2015). Difficult Airway Society 2015 guidelines for management of unanticipated difficult intubation in adults. Br J Anaesth..

[15] Cleophas TJ, Zwinderman AH (2018). Modern Bayesian statistics in clinical research.

[16] Wagenmakers EJ, Morey RD, Lee MD (2016). Bayesian benefits for the pragmatic researcher. Curr Dir Psychol Sci.

[17] Matthews RA (1998). Bayesian statistical methods: what, why–and when. J Altern Complement Med.

[18] Everitt B, Skrondal A. The Cambridge dictionary of statistics. http://www.books24x7.com/marc.asp?bookid=36106 (2010).

[19] Goodman SN (2005). Introduction to Bayesian methods I: measuring the strength of evidence. Clin Trials.

[20] Jack Lee J, Chu CT (2012). Bayesian clinical trials in action. Stat Med.

[21] Ryan EG, Harrison EM, Pearse RM, Gates S (2019). Perioperative haemodynamic therapy for major gastrointestinal surgery: the effect of a Bayesian approach to interpreting the findings of a randomised controlled trial. BMJ Open..

[22] Pearse RM, Harrison DA, MacDonald N, Gillies MA, Blunt M, Ackland G, Grocott MPW, Ahern A, Griggs K, Scott R, Hinds C, Rowan K, Group for the OS (2014). Effect of a perioperative, cardiac output-guided hemodynamic therapy algorithm on outcomes following major gastrointestinal surgery: a randomized clinical trial and systematic review. JAMA..

[23] Grocott MPW, Dushianthan A, Hamilton MA, Mythen MG, Harrison D, Rowan K (2012). Perioperative increase in global blood flow to explicit defined goals and outcomes following surgery. Cochrane Database Syst Rev.

[24] Kruschke JK, Liddell TM (2018). Bayesian data analysis for newcomers. Psychon Bull Rev.

[25] Maitre PO, Stanski DR (1988). Bayesian forecasting improves the prediction of intraoperative plasma concentrations of alfentanil. Anesthesiology.

[26] Motamed C, Devys J-M, Debaene B, Billard V (2012). Influence of real-time Bayesian forecasting of pharmacokinetic parameters on the precision of a rocuronium target-controlled infusion. Eur J Clin Pharmacol..

[27] van den Berg JP, Eleveld DJ, De Smet T, Van Den Heerik AVM, Van Amsterdam K, Lichtenbelt BJ, Scheeren TWL, Absalom AR, Struys MMRF (2017). Influence of Bayesian optimization on the performance of propofol target-controlled infusion. Br J Anaesth..

[28] Vellinga R, Hannivoort LN, Introna M, Touw DJ, Absalom AR, Eleveld DJ, Struys MMRF (2021). Prospective clinical validation of the Eleveld propofol pharmacokinetic-pharmacodynamic model in general anaesthesia. Br J Anaesth.

[29] Colin P, Eleveld DJ, van den Berg JP, Vereecke HEM, Struys MMRF, Schelling G, Apfel CC, Hornuss C (2016). Propofol breath monitoring as a potential tool to improve the prediction of intraoperative plasma concentrations. Clin Pharmacokinet..

[30] Kuck K, Egan TD (2017). Getting the dose right: anaesthetic drug delivery and the posological sweet spot. Br J Anaesth.

[31] Struys MMRF, De Smet T, Versichelen LFM, Van de Velde S, Van den Broecke R, Mortier EP (2001). Comparison of closed-loop controlled administration of propofol using bispectral index as the controlled variable versus “standard practice” controlled administration. Anesthesiol J Am Soc Anesthesiol..

[32] Struys MMRF, De Smet T, Greenwald S, Absalom AR, Binge S, Mortier EP (2004). Performance evaluation of two published closed-loop control systems using bispectral index monitoring: a simulation study. Anesthesiology..

[33] De Smet T, Struys MMRF, Greenwald S, Mortier EP, Shafer SL (2007). Estimation of optimal modeling weights for a bayesian-based closed-loop system for propofol administration using the bispectral index as a controlled variable: a simulation study. Anesth Analg..

[34] De Smet T, Struys MMRF, Neckebroek MM, Van den Hauwe K, Bonte S, Mortier EP (2008). The accuracy and clinical feasibility of a new bayesian-based closed-loop control system for propofol administration using the bispectral index as a controlled variable. Anesth Analg..

[35] Neckebroek M, Ionescu CM, van Amsterdam K, De Smet T, De Baets P, Decruyenaere J, De Keyser R, Struys MMRF (2019). A comparison of propofol-to-BIS post-operative intensive care sedation by means of target controlled infusion, Bayesian-based and predictive control methods: an observational, open-label pilot study. J Clin Monit Comput..

[36] Neckebroek M, Boldingh JWHL, De Smet T, Struys MMRF (2020). Influence of remifentanil on the control performance of the bispectral index controlled Bayesian-based closed-loop system for propofol administration. Anesth Analg.

[37] Kuizenga MH, Vereecke HEM, Struys MMRF (2016). Model-based drug administration: current status of target-controlled infusion and closed-loop control. Curr Opin Anaesthesiol.

[38] Absalom AR, De Keyser R, Struys MMRF (2011). Closed loop anesthesia: are we getting close to finding the holy grail?. Anesth Analg.

[39] Zaouter C, Joosten A, Rinehart J, Struys MMRF, Hemmerling TM (2020). Autonomous systems in anesthesia: where do we stand in 2020? A narrative review. Anesth Analg.

[40] Masui K, Kira M, Kazama T, Hagihira S, Mortier EP, Struys MMRF (2009). Early phase pharmacokinetics but not pharmacodynamics are influenced by propofol infusion rate. Anesthesiology..

[41] Kwon Y, Won J-H, Kim BJ, Paik MC (2020). Uncertainty quantification using Bayesian neural networks in classification: Application to biomedical image segmentation. Comput Stat Data Anal.

[42] Ghahramani Z (2015). Probabilistic machine learning and artificial intelligence. Nature.

